# *In Vivo* Sampling of Intracellular Heterogeneity of *Pseudomonas putida* Enables Multiobjective Optimization of Genetic Devices

**DOI:** 10.1021/acssynbio.3c00009

**Published:** 2023-05-17

**Authors:** Angeles Hueso-Gil, Belén Calles, Víctor de Lorenzo

**Affiliations:** Systems Biology Department, Centro Nacional de Biotecnología-CSIC, Campus de Cantoblanco, Madrid 28049, Spain

**Keywords:** interoperability, *Pseudomonas*, CcaSR system, PleD, biofilm, transposon

## Abstract

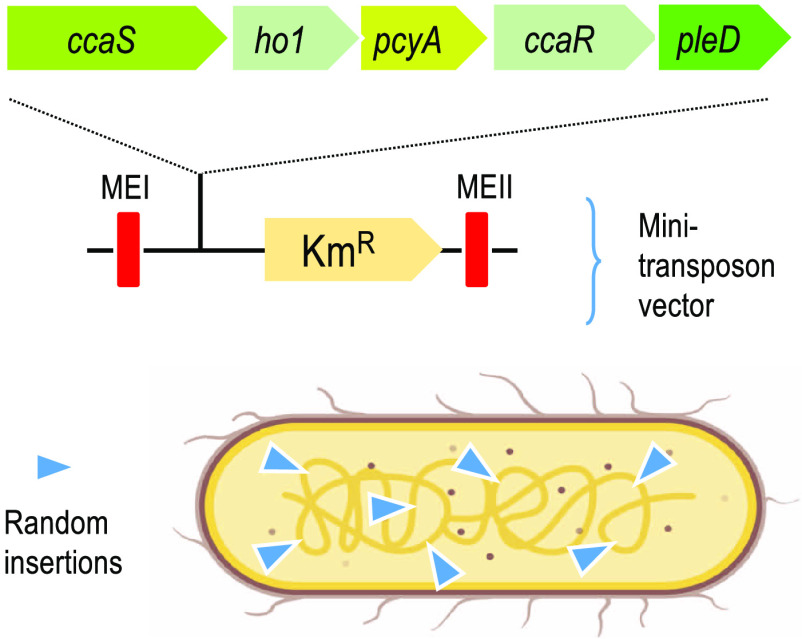

The inner physicochemical heterogeneity of bacterial cells generates three-dimensional (3D)-dependent variations of resources for effective expression of given chromosomally located genes. This fact has been exploited for adjusting the most favorable parameters for implanting a complex device for optogenetic control of biofilm formation in the soil bacterium *Pseudomonas putida*. To this end, a DNA segment encoding a superactive variant of the *Caulobacter crescendus* diguanylate cyclase PleD expressed under the control of the cyanobacterial light-responsive CcaSR system was placed in a mini-Tn5 transposon vector and randomly inserted through the chromosome of wild-type and biofilm-deficient variants of *P. putida* lacking the *wsp* gene cluster. This operation delivered a collection of clones covering a whole range of biofilm-building capacities and dynamic ranges in response to green light. Since the phenotypic output of the device depends on a large number of parameters (multiple promoters, RNA stability, translational efficacy, metabolic precursors, protein folding, etc.), we argue that random chromosomal insertions enable sampling the intracellular milieu for an optimal set of resources that deliver a preset phenotypic specification. Results thus support the notion that the context dependency can be exploited as a tool for multiobjective optimization, rather than a foe to be suppressed in Synthetic Biology constructs.

Engineering live systems with the conceptual and material tools of contemporary Synthetic Biology typically starts with a functional assembly of DNA parts following a rational blueprint. The thereby generated prototypes are then subject to one of more design-build-test-learn cycles until the constructs at stake deliver the right performance *in vivo* upon acquisition of an optimal configuration of working parameters.^1^ The process is currently facilitated by a plethora of computational resources for optimization of such designs.^2,3^ When the construct involves just a few parts, it is growingly possible to precompose specific DNA sequences and then implement them *in vivo* with a high degree of functional predictability. But increasing the number of parts also augments the difficulty of multiparameter adjustment and upfront optimality is difficult to realize.^4,5^ The way out in these cases usually comprises diversification of regulatory sequences (e.g., promoters and intergenic regions) followed by screening or *in vivo* selection of the best performers.^6,7^ This can be made with a large number of strategies that target such sequences and enable the biological system to fluctuate through a solution space until it meets an externally prefixed outcome. Stratagems such as the MAGE-based DIvERGE^8^ or CRISPR-Cas9-based techniques^9,10^ epitomize the said approaches in which diversification and selection cycles solve optimization problems, which are not amenable to explicit calculations with the existing level of knowledge or computation power. These methodologies in fact echo the way extant biological systems find evolutionary solutions to comparable multitiered adaptive challenges. Similar to laboratory setups, variability of control signals for transcription or translation enables exploration of the solution space for bringing about an optimal stoichiometry of each of the products involved.^6,7^ This scenario could be conceptualized as an adjustment of the kinetic constants of the DNA and RNA sequences at stake in their interaction partners (RNAP, ribosomes), which predictably change when the nucleotide sequence involved varies as well. Note, however, that changes in such interactions could also result from variations in local concentrations and availability of small molecules (precursors, metabolites, effectors) at the site of the event.^11−14^ This in turn elicits differences in the observable activity of the device.

We have recently reported that—similar to other bacteria—the intracellular milieu of the soil dwelling and Synthetic Biology chassis *Pseudomonas putida* is by no means homogeneous.^15,16^ Instead, various components of the gene expression flow (RNAP, ribosomes, transcripts) seem to be segregated in specific locations with respect to the nucleoid DNA. This scenario necessarily implies that not every DNA segment of the chromosome is exposed to the same availability of components of the molecular machinery for expression. Furthermore, proximity to the origin of replication^17−19^ and inclusion in different chromosomal superloops^20−22^ may enter considerable differences in protein–DNA interaction parameters.^12,23^ Finally, every chromosomal location is submitted to a very variable degree of readthrough and pervasive transcription.^24^ While the ensuing context sensitivity has been generally considered a limitation for sound biological engineering, we wondered whether such intracellular heterogeneity could be instead leveraged to optimize the performance of genetic constructs.^13,25−28^ Not by diversifying regulatory sequences but by physically sampling the three-dimensional (3D) parameter landscape that different chromosomal locations are exposed to.

In order to address this question, we have utilized a complex synthetic device with an easy phenotypic readout (i.e., biofilm formation) as a probe of multiobjective optimization upon random insertion in diverse sites. Adopting such a composite reporter, which depends not only on gene expression but also on availability of metabolic resources, enabled us to go beyond the mere variation of transcription/translation along the chromosome. As explained below, this approach enabled *P. putida* cells to survey and eventually find the optimal set of parameters for sticking to surfaces upon exposure to green light.^7^ On this basis, we argue that intracellular granularity and context-dependent changes of interaction parameters are more an asset than a liability for construction of effective genetic devices. Furthermore, the data below strengthen the notion that the spatial location of the DNA sequences in the 3D architecture of the chromosome is one more evolutionary mechanism—different from straight mutations—through which bacteria resolve adjustment of biological activities to given environmental challenges.

## Results

### Rationale for Leveraging Intracellular Heterogeneity for Device Optimization

**Figure 1:**
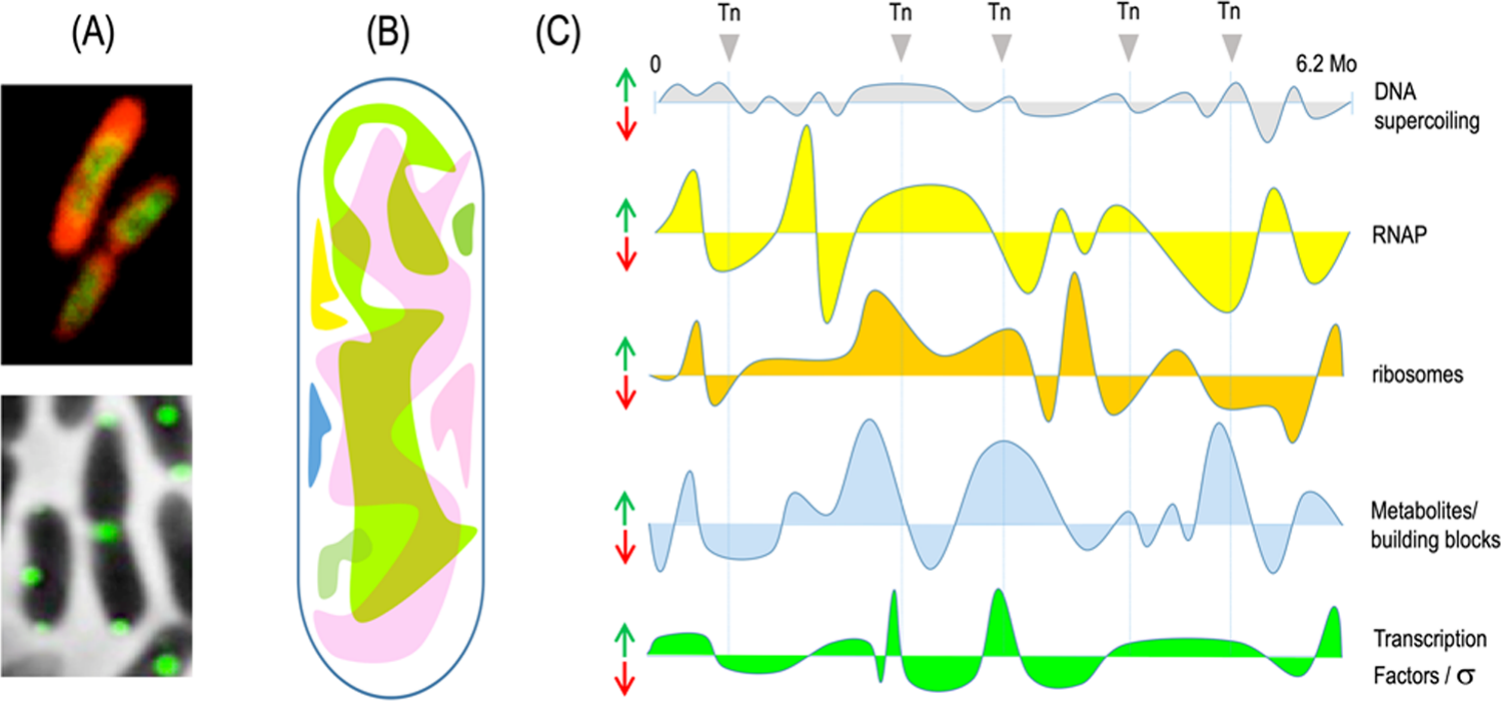
Intracellular heterogeneity of the *P. putida* cytoplasm and its consequences. Resources for transcription and translation cannot be homogeneous due to differences in DNA folding and unequal 3D distribution of the gene expression hardware. Diverse physicochemical properties of each intracellular address and uneven localization of polymerases, transcription factors, ribosomes, and metabolites along with variable DNA supercoiling conform a landscape^68^ that affects the final performance of the implemented device depending on the spot of insertion. (A) Top: fluorescence microscopy picture of the distribution of RNA polymerase (green) and ribosomes (red), reproduced with permission from reference (15) (Copyright 2019 John Wiley and Sons). Bottom: Green dots on the bottom picture mark localization of pWW0 plasmid of *P. putida* mt-2, reproduced from reference (12) (Copyright 2017 American Chemical Society). (B) Recreation of the uneven distribution of cellular components inside cells. (C) Insertions of a genetic device in different chromosomal locations are exposed each to different concentrations of resources, giving as a result a diverse molecular landscape for transcription, translation, and protein activity.

The background of the results presented in this work takes on [i] that the 3D distribution of chromosomal DNA in the bacterial cell provides a physical scaffold that assigns a specific address to the sequence of every gene within the cytoplasmic space^12,13,29^ and [ii] that the relative position of the chromosome in respect to the rest of the cell’s shape is largely kept in all growing conditions.^15^ Given the heterogeneity of the intracellular milieu, one can then entertain that every DNA segment of the chromosome is plausibly exposed to different levels of the components of the gene expression flow as well to diverse degrees of supercoiling and exposed to readthrough transcription from nearby promoters (Figure 1). This creates in turn a diverse molecular environment in which distinct genomic sites face very different levels of effectors, building blocks, and interaction partners.^25,30^ This can of course vary also when the same genes move to another host. We thus entertained that chromosomal localization offers a landscape of solutions able to deliver specific levels and attributes of expression to a designed construct.

**Figure 2:**
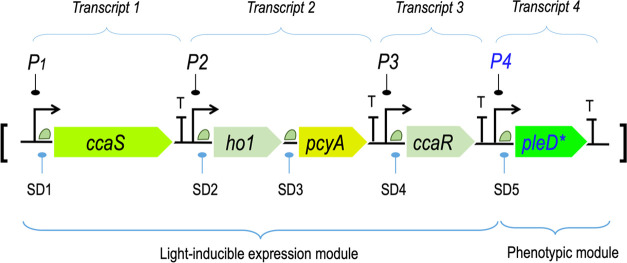
Organization of the OPT·FILM device. The genetic construct leveraged for this work (not to scale) is composed of a module for sensing green light (encoding sensor CcaS, PCB enzymes Ho1 and PcyA, and transcriptional regulator CcaR) adjacent to the phenotypic module, which, in this case, corresponds to the cdGMP and the biofilm-promoting PleD* enzyme. Genes are transcribed as separate units (except *ho1* and *pcyA* that form a two-gene operon) punctuated by regulatory regions (promoters and Shine–Dalgarno sequences^7^).

In order to bring these speculations to a tractable experimental setup, we picked the optogenetic device^31^ sketched in Figure 2 that we call OPT·FILM. This is a 5-gene synthetic DNA segment consisting of two modules. The first [*ccaS·ho1/pcyA·ccaR·P_cpcG_*_2-172_ →] bears the ORFs encoding the green light sensor (CcaS) and its cognate regulatory counterpart (CcaR) of *Synechocystis* PCC6803 separated by the genes of the two enzymes (Ho1 and PcyA) for production of phycocyanobilin (PCB). In this system, PCB acts as a cofactor that lets the CcaS sensor to autophosphorylate and later transfer this phosphoryl group to CcaR. In turn, the phosphorylated CcaR then acts as a transcription factor (TF) that recognizes the *P*_cpcG2_ promoter and activates expression of downstream genes. In this work, the sequence of the CcaR-activated promoter *P*_*cpcG*2-172_ was assembled in an outward-looking orientation, a modified version that shows a higher dynamic range. Variants of this [*ccaS·ho1/pcyA·ccaR·P_cpcG_*_2-172_ →] device have been exploited in the past to engineer light-responsive gene expression in diverse bacterial hosts, including *P. putida.*(7,32,33) The second component of OPT·FILM was a DNA sequence encoding a modified version of PleD, a diguanylate cyclase of *Caulobacter sp.*(34−36) Variant PleD* has 4 point mutations that make the enzyme constitutively able to produce high intracellular levels of c-di-GMP.^37−39^ Elevated concentrations of this second messenger trigger production of a sticky external matrix composed of proteins, exopolysaccharides, and DNA that results in biofilm formation.^40^ The expected operation of the OPT·FILM device was thus taking green light as the input and fostering biofilm formation as the desired phenotypic output.

**Figure 3:**
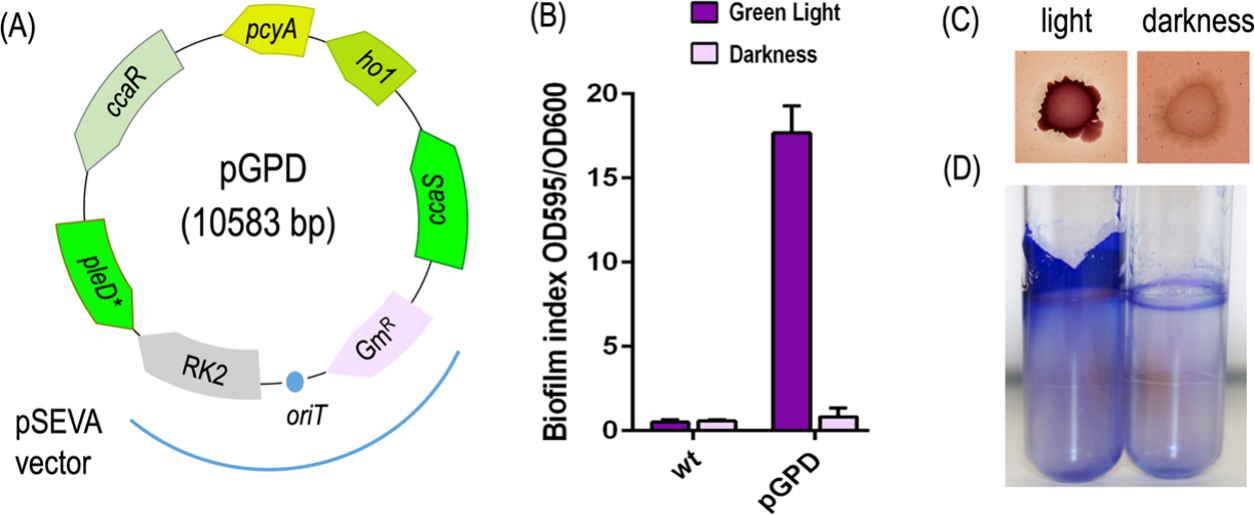
Benchmarking the OPT·FILM device in *P. putida*. (A) pGPD plasmid stems from the previously described pGreenL^7^ by replacing the GFP reporter sequence by the pleD* gene, which encodes a superactive diguanylate cyclase for triggering a biofilm formation phenotype. (B) *P. putida* KT2440 cells bearing or not pGPD plasmid were tested for biofilm formation upon exposure to green light. Cells carrying the plasmid displayed a clear light-inducible biofilm formation as quantified with the crystal violet assay. (C) Congo Red/Coomassie-stained colonies of the same strains exposed or not to green light. (D) Visual inspection of cell growth on the walls of the same cultures.

In order to benchmark the OPT·FILM device, plasmid pGPD (Figure 3A) was built in which the complete construct was assembled in low-copy number vector pSEVA621 (Supporting Information Table S1) and placed in *P. putida* KT2440. The transformants were then cultured—under either green light or darkness—in microtiter plates with no shaking for 16 h. After this period, biofilm was measured with the crystal violet test (see Materials and Methods), a procedure that has become the test of choice for quantifying biofilm formation in virtually all types of bacteria. While it is true that the measurable readout is indirect, there is a robust quantitative relation between dye retention in the microtiter plate walls, the biofilm matrix, and the number of cells trapped in it. As shown in Figure 3B, cells of light-induced cultures adhered surfaces to a much higher level than the cultures kept in the dark. The same became apparent in other two tests for gross estimation of biofilm formation: cellulose accumulation in agar surfaces as detected with Congo Red (Figure 3C) and adhesion to the walls of plastic tubes in liquid cultures with crystal violet (Figure 3D). Taken together, these data indicated that the properties and parameters of the [*ccaS·ho1/pcyA·ccaR·P_cpcG_*_2-172_ →] device gauged earlier with GFP technology^7^ were grossly kept when the fluorescent reporter was replaced by PleD* in a plasmid vector. But what happens when the very same genetic module goes into different chromosomal locations?

### Inserting OPT·FILM through the *P. putida* Chromosome

**Figure 4:**
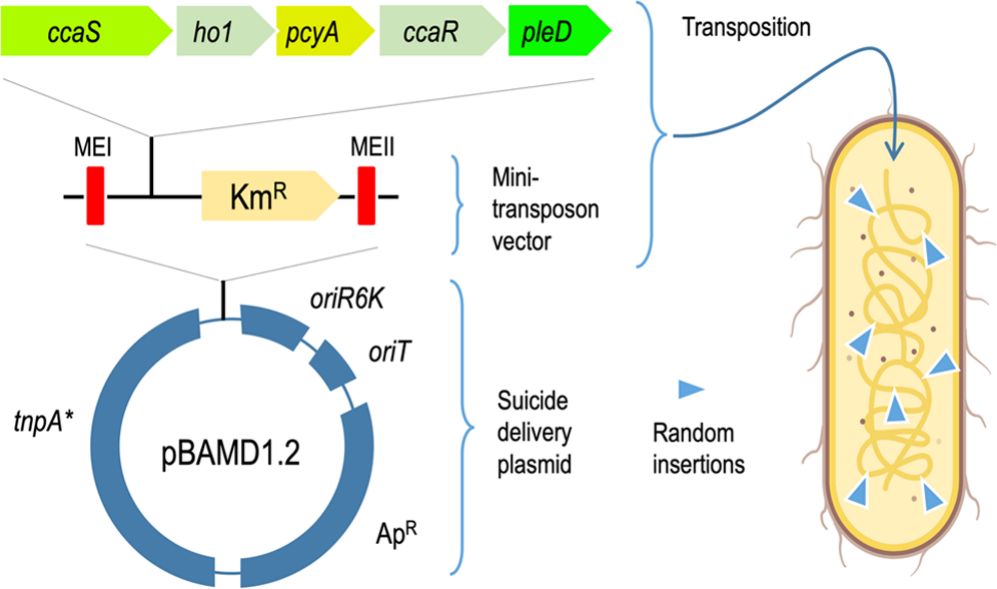
Genetic strategy for random sampling of chromosomal sites of *P. putida* with mini-Tn*5* [OPT·FILM]. The OPT·FILM cassette bearing the light-inducible device for biofilm formation (top left) was cloned inside the Km^R^ mini-Tn*5* transposon vector borne by delivery plasmid pBAMD1.2 (sketched bottom left), flanked by the MEI and MEII regions (ME: mosaic ends for transposase recognition). Upon conjugal mobilization of the resulting construct toward *P. putida* (whether the wild-type strain or its *wsp* derivative), the device becomes randomly inserted through the chromosome of the target bacterium (right). This results in placing the OPT·FILM segment in a variety of chromosomal sites, themselves located at different locations of the intracellular 3D space.

The signal processing through the OPT-FILM device involves 4 transcriptional units—each run by a separate promoter—and 5 ribosomal binding sites, which determine the translation initiation of proteins, which need of course being eventually expressed in an active form and a specific stoichiometry. Selection of the most favorable configuration of the [*ccaS·ho1/pcyA·ccaR·P_cpcG_*_2-172_ →] part of the device was done by making intergenic regions (SDs, promoters) to fluctuate with MAGE technology followed by selection of the best performers.^7^ The starting point is therefore an expression system that was optimized for performance in *P. putida* in the frame of a low-copy number plasmid. RK2-based replicons devoid of a Par system seem to move freely through various locations of the cell cytoplasm^41^ and therefore they might be less influenced by intracellular 3D heterogeneity. To investigate how the same device performed when expressed from different intracellular addresses, the same OPT·FILM segment was cloned inside the mini-Tn5 transposon of suicide delivery plasmid pBAMD1.2.^42^ As shown in Figure 4, this vector enables random and unique chromosomal insertions of any cargo placed between the ME ends of a Km^R^ mobile element. The whole can then become stably implanted in virtually any place of the chromosome and its cargo DNA consigned to a distinct spot of the 3D cytoplasmic space. We hypothesized that such a strategy enabled physical exploration of the parameter landscape and thus finding locations, which provided the right constellation of resources for bringing about optimal expression of the light-induced phenotype.

In the first series of experiments, the mini-Tn5 [OPT·FILM] transposon was delivered to the reference strain *P. putida* through a standard tripartite mating with the donor *Escherichia coli* DH5α λ*pir* (pBAMD1.2-OPT·FILM) cells and helper strain *E. coli* HB1010 (pRK600). Mating mixtures were subsequently plated on Petri dishes with minimal agar citrate and Km for selection of exconjugants bearing insertions of the mobile element in the target *P. putida* strain. Under these conditions, most insertions of the hybrid mini-Tn5 occurred through a genomic segment closer to the origin of replication (see Materials and Methods). A library of approximately 200 distinct Km^R^*P. putida* clones, each hypothetically bearing a different insertion, was thereby generated. A subset of randomly picked, healthy-looking clones were then reisolated and tested for sensitivity to ampicillin at 500 μg/mL. This verified occurrence of clean insertions of mini-Tn5 [OPT·FILM] instead of illegitimate cointegration of the whole delivery plasmid pBAMD1.2. 47 of such Km^R^ Ap^S^ exconjugants were then subject to further analyses as described next.

### Characterization of Mini-Tn5 [OPT-FILM]-Inserted *P. putida* Clones

Following the procedures above, each of the thereby isolated clones was tested for chromosomal location, growth traits, and ability to form biofilm when exposed (or not) to green light. Supporting Information Table S2 lists the genomic sites, where mini-Tn5 [OPT·FILM] insertions were unequivocally found. Most of them had little or no impact on gross growth parameters in LB (Supporting Information Figure S1), i.e., attained the same cell density after 23 h. Yet, others were affected by the insertion and reached a lower OD_600_ value. This did not come as a surprise, as insertion of the mobile device in these cases occurred in housekeeping genes important for cell physiology, such as adenosyl homocysteinase (clone F2), 16S ribosomal RNA (G6), ATP-dependent RNA helicase (A12), and a 50S ribosomal protein (C8).

**Figure 5:**
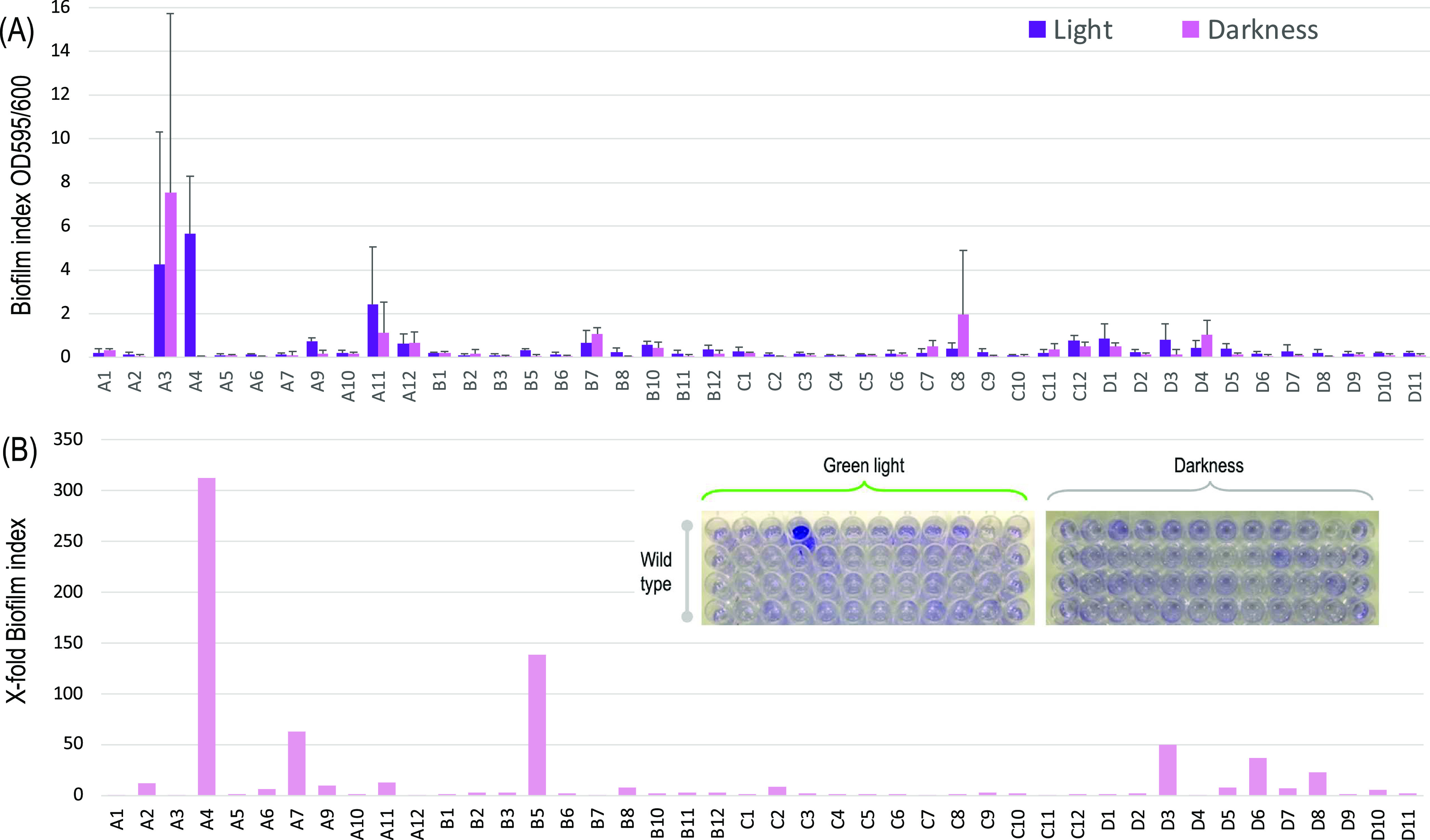
Biofilm formation by *P. putida* KT2440 clones inserted with mini-Tn*5* [OPT·FILM] at diverse chromosomal locations. The biofilm index for every clone of a transposon library sample is shown. This index is calculated by dividing the absorbance measurement for biofilm at 595 nm after crystal violet staining by the absorbance at 600 nm of cultures at the beginning of the protocol. Biofilm index is a quantitative value for biofilm formation. X-fold biofilm index represents the biofilm index of a culture exposed to light divided by the biofilm index of the same culture kept in darkness. Differences in the behavior of each variant (named after the well in the plate where they were growing) can be noticed by comparing biofilm formation in plates exposed or not to green light. The plots display the results of three biological replicates. (B) Same data represented as X-fold induction. For example, strain A4 of this group of clones looks like a good case of optimized performance (located by PP_5368, Supporting Information Table S2).

For studying biofilm formation in response to green light, 1 μL of an overnight culture of each of the 47 clones was separately inoculated in 200 μL of M9 citrate medium in microtiter plates and incubated without shaking for 16 h either under complete darkness or exposed to the green light emitted by the LED panel described in Materials and Methods. Adhesion to surfaces was then quantified with the standard crystal violet test.^43^ The results shown in Figure 5 exposed how the same genetic device behaved very differently depending on the chromosomal location from which it was expressed. Despite the limited number of clones analyzed, even such a small sample covered a considerable collection of phenotypes. In general, attachment to surfaces (whether under green light or not) was significantly lower in the cells bearing the OPT·FILM device in the chromosome as compared to biofilm formation of *P. putida* KT2440 transformed with plasmid pGPD (Figure 3), as considerable differences were found in their responsiveness to light, net adhesion to the plastic coatings, and on/off ratios. As listed in Supporting Information Table S2, insertions occurred either in or in close proximity of a whole variety of functional genes. It has been argued that chromosomal regions encoding ribosomal components have higher local levels of RNAP and thus constitute good sites for locating heterologous genes.^23^ Various insertions in at least one of such segments (e.g., G1, H1, and A2) produced however a modest biofilm-forming phenotype, thereby suggesting a role for other ingredients of the gene expression flow as well. In other cases, exposure to green light conspicuously downregulated rather than upregulated attachment to surfaces. Finally, some insertions (e.g., A4) displayed a sort of ideal behavior, i.e., a very low basal level of expression in darkness and quite noticeable surface attachment under illumination. This insertion was located in gene PP_5368 (Supporting Information Table S2) disrupting a major facilitator superfamily (MFS) transporter, responsible for the transfer of small solutes through a lipid membrane. Among the best performers, none of them were found close to ribosomal gene operons.^23^ However, some of the clones with a lower induction were in those locations. The key for a good on/off ratio of the OPT·FILM device for biofilm formation is likely to reside in a fine balance of its components and not in the mere overexpression of them.

In sum, although the reduced number of insertions by no means covered a wide range of sites of the chromosome, the results clearly illustrated that the location had a dramatic impact in the phenotypic outcome of the device. While some of the differences could be attributed to a positive or negative influence of readthrough transcription from promoters adjacent to the site of insertion, this explains neither the abundance of low-biofilm clones nor the reverse regulation of some isolates (e.g., A3, C8). Since attachment to surfaces seems to ultimately depend on intracellular levels of cdGMP, we wondered whether the native regulatory network for production of such a signal molecule could interfere with the overimposed OPT·FILM construct. We thus re-examined the same issue on a *P. putida* strain entirely disrupted in the endogenous surface-detecting, cdGMP-dependent mechanism.

### Knocking-In the OPT·FILM Device in *wsp*-minus Strains

Similarly to other *Pseudomonas* species, the *wsp* operon of *P. putida* encodes a complete signal transduction apparatus consisting of a 7 protein transmembrane complex, which translates collision of bacteria with solid items into activation of a cognate, major diguanylate cyclase that produces cdGMP, thereby triggering biofilm formation.^44^ Deletion of the whole *wsp* cluster in *P. putida* results in cells with impaired ability to form biofilm.^45^ Δ*wsp* cells thus provide a cleaner physiological background for inspecting the phenotypes of mini-Tn5 [OPT·FILM] insertions. On this basis, we repeated the same transposon delivery protocol employed before on a *P. putida* Δ*wsp* strain known to be deficient in biofilm formation.^45^ Under the circumstances, the OPT·FILM device is expected to take over the job of producing cdGMP upon surface contact and altogether replace the triggering signal by a different input (i.e., green light).

**Figure 6:**
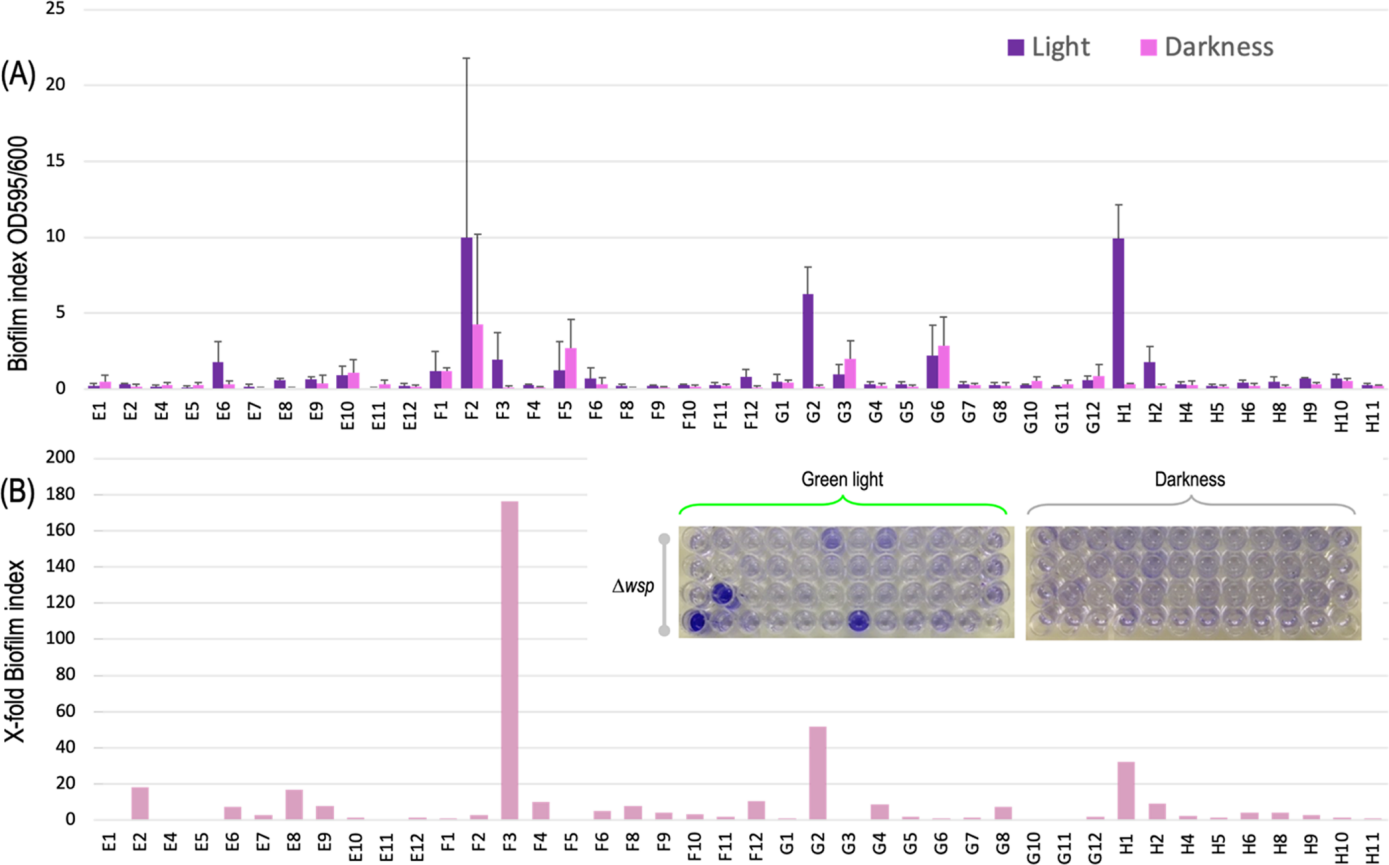
Biofilm formation by *P. putida* KT2440 *wsp* clones inserted with mini-Tn*5* [OPT·FILM]. Similar experiment to that shown in Figure 5 but carried with *P. putida* KT2440 *wsp* as transposon recipient. (A) Biofilm index of three biological replicates. (B) X-fold induction. This library showed a higher variability of biofilm-forming phenotypes (see text for Discussion).

The result of the operation was again a library of Km^R^ Ap^S^ exconjugants. As before, a sample of healthy-looking clones bearing single insertions was picked for determining chromosomal locations of the OPT-FILM segment (Supporting Information Figure S2 and Table S3), their effect on growth inspected (Supporting Information Figure S1) and their biofilm production phenotype with and without green light quantified (Figure 6). Despite the limited number of clones examined, a larger portion of isolates were able to attach to surfaces when microtiter plates were grown under a 520 nm wavelength as compared to those kept in the dark. And within those, we again observed a whole variety of behaviors from high but unregulated biofilm production (e.g., F2 insertion) to relatively modest but highly light-responsive attachment (F3 clone) to no-attachment at all. Interestingly, the F3 insertion was found within the intergenic region PP_0133-PP_0134 (Supporting Information Table S3). PP_0133 encodes the transcriptional regulator AlgB, linked to synthesis of alginate, the production of which has a role in biofilm formation in mildly water-depleted environments.^46^ Although we do not know whether transcription and expression of this gene were directly affected by the insertion, its proximity to F3 may not be alien to the phenotype observed. Other than this location, perusal of the insertion sites did not shed much light on the basis of these phenotypes, as so many local factors can influence eventually the display of the observable trait. But the same results also accredited that exposure of the OPT·FILM device to the physicochemical settings of disparate chromosomal locations caused in turn a variety of biofilm-related displays. Again, there was little correlation between optimized biofilm production/regulation and insertions in sites reported earlier to facilitate high levels of heterologous gene expression.^23,47^ Only one good-performing clone (H1) had OPT·FILM inserted by the ribosomal gene PP_23SA,^23^ while the others with valid phenotypes occurred in other locations. Also, the data of Figure 6 suggested that the native *wsp*-determined surface adhesion program of *P. putida* becomes entirely submitted to the artificial control of cdGMP production knocked-in with the optogenetic device.

## Discussion

That different locations of the bacterial chromosome give rise to very different levels of expression of heterologous genes has been observed for a long time and often attributed to differential supercoiling^48−50^ or proximity to the origin of replication.^17−19^ However, the 3D heterogeneity of protein distribution^51^ along the genome (including RNAP^52^) and the patchy intracellular allocation of ribosomes,^53^ metabolites, and pathways^54,55^ produce a much more variable molecular environment for every chromosome site than anticipated before.^56^ The synthetic optogenetic device OPT·FILM on which this work is based was first optimized *in vitro* to exhibit a present input (green light) to output (biofilm formation) transfer function in *P. putida* when expressed from a low-copy number plasmid (Figure 3). Yet, the data above shows that the same device dramatically diversifies its phenotypic outcome when placed in different chromosomal locations. We attribute such variations to the molecular heterogeneity of the bacterial cytoplasm, which results in a landscape of dissimilar resources for the gene expression flow (ribosomes, RNAP, metabolic availability) at given locations of the 3D space. Such physicochemical unevenness of the cell inside concurs with proximal setting-dependent effects (e.g., local supercoiling, readthrough transcription) at the sites of chromosomal insertion, which can modify vicinal intermolecular interactions. Finally, insertions of the mini-Tn5 [OPT·FILM] transposon can hit native functions that either decrease or enhance the observable phenotype. Because of such hypervariability, random insertions of functional DNA segments in the host genomes may afford exploration of a much denser solution space for multiobjective optimization of genetic devices than that achieved with combinatorial libraries of regulatory parts.

One important aspect of the experiments conducted is the likely cell-to-cell variation in intracellular 3D granularity, given that clones were interrogated at a whole population level. While the phenotype of different insertions was maintained through three biological replicates generated on different days (suggesting that the main repeatability from the insertion at a locus comes from its physical location in the genome rather than the 3D milieu of proteins, factors, and metabolites around it), our results did not *directly* prove a heterogeneous availability of resources. However, some data can be better comprehended under this light. For instance, some insertions close to ribosomal genes did not produce regulated biofilm formation (Supporting Information Tables S2 and S3). This suggests that chromosomal locations might be rich in some components of the gene expression flow, such as RNAP and ribosomes but not in the metabolic building blocks required for biofilm formation. This result challenges the conventional wisdom of targeting heterologous expression to such chromosomal sites, which are high in transcriptional and translational resources. Full display of a complex engineered phenotype, such as biofilm formation in response to light, is not solely about producing the proteins involved but also requires tuning their biochemical activities to the physiological background, as well as providing metabolic precursors for synthesizing, for example, chromophores for the correct function of the light-responsive system and extracellular polymeric substances for surface attachment.

Under this light, the wealth and variety of molecular contexts available in each 3D chromosomal spot can be leveraged to become a phenomenal asset for adjusting the boundaries and parameters of an engineered function in a fashion that possibly resembles a natural mechanism to the same outcome. As a matter of fact, the physical location of genes in the bacterial chromosome seems not to be casual^57−59^ but instead likely to reflect one more molecular stratagem for cracking otherwise intractable adaptation challenges. In sum, the data above adds to the growing evidence indicates that genetic context effects can override *cis* regulatory elements,^60^ pinpointing such context variability as a principal mechanism to evolutionarily optimize input–output functions *in vivo*.

## Materials and Methods

### Strains, Media, and Growth Conditions

The list of strains and plasmids used in this work is indicated in Supporting Information Table S1. Unless otherwise specified, bacteria were grown at 37 °C for *E. coli* strains and 30 °C for *Pseudomonas* in LB medium prepared as either liquid cultures or 1.5% agar plates. Alternatively, 200 μL of M9 minimal medium supplemented with 0.2% (v/w) citrate was inoculated in 96-well microtiter plates and grown at the conditions noted. When required, antibiotics were added to the cultures or plates at the following concentrations: gentamicin (Gm) 10 μg/mL, kanamycin (Km) 50 μg/mL, and ampicillin (Ap) 150 μg/mL for *E. coli*, while 500 μg/mL was the concentration when the resistance was tested in *P. putida* in order to exclude illegitimate insertions of the pBAMD backbone.

### Plasmid Construction

The low-copy number Gm^R^ plasmid bearing the complete OPT-FILM device (Figure 3A) was built by replacing the *GFP* insert of pGreenL^45^ (Figure 3A, Supporting Information Table S4) by the pleD* gene, which encodes a constitutively active variant of a major dcGMP cyclase of *Caulobacter crescentus*. To this end, three DNA segments were prepared and Gibson-assembled *in vitro* as follows. The 1365 bp fragment bearing pleD* was first amplified from plasmid pPleD* using primers PleD-F and PleD-R (Supporting Information Table S3). A second 3640 kb DNA segment was PCR-ed up from pGreenL with primers GSB9/GFP-F and CcaSR-1R covering resistance and origin of replication that came from the pSEVA621 backbone.^61^ Finally, the rest of the construct consisted of a third 5578 kb segment also amplified from pGreenL with CcaSR-1F and GSB9/GFP-R. This resulted in a DNA piece, which contained the main components of the CcaSR system ([*ccaS*·*ho1/pcyA*·*cca*R·*P*_*cpcG*2-172_]). The three segments were then mixed and assembled *in vitro* with a standard isothermal assembly protocol,^62^ the reaction electroporated in *E. coli* DH10B+ cells^63^ and next selected in LB-Gm plates. A few colonies were picked for further analyses, and accuracy of DNA constructs was verified by complete DNA sequencing. The correct plasmid was named pGPD (sketched in Figure 3A; Genebank accession number OQ548061) and was used thereafter as a reference for the rest of the work. In order to place the thereby assembled OPT·FILM segment (Figure 2) in a mobile element, two PCR products encompassing the whole sequence were generated from pGPD. The first DNA fragment, amplified with primers CcaSR/pBAM-F and CcaSR-2R, covered 3496 bb of the device spanning *ccaS* and the majority of the gene *ho*1. The second DNA segment, carrying the rest of the OPT·FILM sequence, was obtained with primers CcaSR-2F and CcaSR/pBAM-R (Supporting Information Table S4). These two PCR products were then mixed with EcoRI/HindIII-digested mini-Tn5 delivery vector pBAMD1.2 (Supporting Information Table S1) and subject to a Gibson assembly.^62^ The reaction mix was in this case electroporated in *E. coli* DH5α λ*pir* to secure replication of the *ori* R6K of the vector. The plasmids carried by few Ap^R^ Km^R^ clones were analyzed, and the resulting verified construct named pBAMD1.2 [OPT·FILM], in which the device *ccaS*·*ho1/pcyA*·*cca*R·*P*_*cpcG*2-172_ → pleD* (Figure 2) was placed inside the Km^R^ mini-Tn5 transposon vector borne by pBAMD1.2 (sketched in Figure 4).

### Construction of Mini-Tn5 [OPT-FILM] Insertion Libraries in *P. putida*

In order to insert the mobile element encoding the light-responsive biofilm formation device through the chromosomes of either the wild-type strain KT2440 or its adhesion-deficient derivative lacking the *wsp* cluster (Supporting Information Table S1), triparental conjugation mixes were set up, as described by Martínez-García et al.,^64,65^ with some modifications. Separate cultures of the donor strain *E. coli* DH5α λ*pir* (pBAMD1.2 [OPT-FILM]), recipient *P. putida* cells, and helper *E. coli* HB101 (pRK600) were separately cultured overnight in LB medium with antibiotics when necessary. The following day, cultures of each of the strains were adjusted to an OD_600_ of 1.0 and100 μL of each was put together (in a ratio 1:1:1), centrifuged and resuspended in a 20 μL drop of 10 mM MgSO_4_. This process was done in 10 replicates for both the wild-type *P. putida* KT2440 and its *wsp* mutant derivative. Suspensions were then placed on top of LB agar 1.5% (v/w) plates and incubated at 30 °C for 5 h. After the incubation period, biomass patches from each mating mix were resuspended in 1 mL of 10 mM MgSO_4_ and plated on M9 citrate with Km to select for *P. putida* clones, which had acquired the mini-Tn5 [OPT·FILM] transposon. A library of approximately 200 potentially inserted clones was thereby generated, regardless of the *P. putida* strain used. Individual colonies were streaked out and their sensitivity to β-lactams tested on LB plates with Ap (500 μg/mL) and Km (50 μg/mL) for distinguishing *bona fide* transposon insertions (Km^R^ Ap^S^ clones) from illegitimate integration of the delivery plasmid in the *P. putida* chromosome (Km^R^ Ap^R^ colonies). Approximately 25% of the Km^R^*P. putida* exconjugants turned out to be authentic mini-Tn5 insertions. A sample of 47 healthy-looking colonies from each mating was then inspected for determination of the chromosomal sites, where the OPT·FILM device had been incorporated. To this end, the DNA sequence adjacent to the site of insertion was amplified as described,^42,66^ the resulting PCR fragments were purified, their DNA sequence was determined (Macrogen, Inc.), and the genomic location of transposons identified by BLAST search using www.pseudomonas.com database.

### Biofilm Formation

Quantification of attachment of *P. putida* cells to the plastic surfaces of microtiter plates was done with the classic crystal violet protocol of O’Toole et al.^43^ with some modifications for inspecting responsiveness to light. 1 μL of a preculture of each of the clones to be tested grown overnight in liquid M9 citrate was used to inoculate 200 μL of fresh medium in 96-sample microtiter plates (transparent polystyrene flat bottom plates; Avantor Sciences https://es.vwr.com/). The thereby prepared cultures were then placed on top and at a distance of 7 cm of a green light source of 520 nm. For this, we used a computer-controlled RGB LED panel matrix connected to an Arduino device with the specifications and settings described previously.^7^ Control cultures were kept in the darkness covered with aluminum foil. Whether illuminated or not, plates were incubated for 16 h at 30 °C with no shaking, after which they were processed for determination of biofilm formation index (BFI) and surface attachment inducibility as explained earlier.^43^ For *P. putida* carrying plasmid pGPD, this protocol was applied to three biological replicates, each with three technical replicates. In the case of clones of the OPT·FILM libraries, three biological replicates were processed, each with two technical replicates. Inspection of cellulose export in colonies or patches as a gross proxy of biofilm-producing regime was run in Congo red agar plates as described.^67^

## Supporting Information Available

Tables with description of strains and plasmids (Table S1), insertions in wild-type strain (Table S2), insertions in *wsp* strain (Table S3) and PCR primers (Table S4). Figures with growth curves (Figure S1) and insertion location (Figure S2) (PDF)
