# Supplementary material for: In Vivo Sampling of Intracellular Heterogeneity of Pseudomonas putida Enables Multiobjective Optimization of Genetic Devices
Source: ACS Synth Biol. 2023 May 17;12(6):1667–76. doi: 10.1021/acssynbio.3c00009 (PMC10278179; doi:10.1021/acssynbio.3c00009)
Supplement: Supplementary file 1 — sb3c00009_si_001.pdf [file sb3c00009_si_001.pdf]

Supplementary Information

to

In vivo sampling of intracellular heterogeneity of *Pseudomonas putida* enables multi-objective optimization of genetic devices

by

Angeles Hueso-Gil[ᘓ], Belén Calles and Víctor de Lorenzo[*]

Systems Biology Department, Centro Nacional de Biotecnología-CSIC, Campus de Cantoblanco, Madrid, 28049, Spain.

[ᘓ] Current address: Centro de Biotecnología y Genómica de Plantas, Universidad Politécnica de Madrid, Instituto Nacional de Investigación y Tecnología Agraria y Alimentaria, Pozuelo de Alarcón, 28223 Madrid, Spain

Supplementary Table S1. Strains and plasmids used in this work.

Escherichia coli		
**Strain**	**Description**	**Reference**
DH10B$^+$	F$^-$, *mcrA, Δ(mrr-hsdRMS-mcrBC), Φ80dlacZΔM15, ΔlacX74, endA1, recA1, deoR, Δ(ara,leu)7697, araD139, galU, galK, nupG, (Str^R), rpsL* λ$^-$	1
DH5α λ*pir*	λ*pir* phage lysogen of DH5α, π$^+$	Lab collection
HB101	F–, *thi-1, hsdS20* (rB–, mB–), *supE44, recA13, ara-14, leuB6, proA2, lacY1, galK2, rpsL20* (str^R), *xyl-5, mtl-1.*	2
Pseudomonas putida		
KT2440	Prototrophic, wild-type strain derived of *P. putida* mt-2 without pWW0 plasmid	3
KT2440 Δwsp	KT2440 derivative with a full deletion of the *wsp* operon	4
**Plasmids**	**Description**	**Reference**
pPleD*	Ap^R, *oriV* pMB1, pUC57 derivative, *pleD** diguanylate cyclase from *Caulobacter crescentus* with four point mutations that make it constitutively active [5, 6]	GeneCust
pGreenL	Gm^R, oriV RK2, [*ccaS•ho1/pcyA•cca*R• $P_{cpcG2\text{-}172}$ → *msf GFP*]	7
pGPD	pGreenL derivative, *msfGFP* replaced by pleD* encoding a constitutively active version of diguanylate cyclase of *C. crescentus.*	This work, Genebank OQ548061
pBAMD1.2	mini-Tn*5* delivery plasmid; *oriV* R6K; Ap^R Km^R	8
pBAMD1.2 [OPT•FILM]	pBAMD1.2 inserted with [OPT•FILM] cassette from pGPD	This work
pRK600	*tra*$^+$ *mob*$^+$ Cm^R *oriV* ColE1 helper plasmid transformed into *E. coli* HB101 to assist conjugation between donor and receiver strain.	9

Supplementary Table S2. Localization mini-Tn*5* [OPT•FILM] insertions in the genome of *P. putida* KT2440 wt. Clones without a specific location or having a fraction of the pBAMD1.2 backbone were classified as illegitimate insertions. Genome coordinates and gene number from www.pseudomonas.com

Clone	Genome coordinate	PP number	Strand	Gene name and putative function
A1	728862	PP_0623	+	*rluD*, 23S rRNA pseudouridine(1911/1915/1917) synthase
B1	264917	PP_0214	+	*gabT*, 5-aminovalerate aminotransferase DavT
C1	951524	PP_0813	+	*cyoB*, cytochrome bo terminal oxidase subunit I
D1	5748159	PP_5044	-	*typA*, ribosome associated GTPase
A2	175069	PP_23SA	+	PP_23SA, rRNA
B2	4657365	PP_4121	+	*nuoC*, NADH-quinone oxidoreductase subunit C/D
C2	6112217	PP_5363	-	PP_5363, hypothetical protein
D2	5657977	PP_4966	+	*sahR*, methionine metabolism transcriptional regulator
A3	6051271	PP_5303	+	*ridA*, aminoacrylate/iminopropionate hydrolase/deaminase
B3	5546480	PP_4880	-	*rnr*, exoribonuclease R
C3	728512	PP_0623	+	*rluD*, 23S rRNA pseudouridine (1911/1915/1917) synthase
D3	5538631	PP_4872	-	PP_4872, hypothetical protein
A4	6119043	PP_5368	-	PP_5368, MFS transporter
C4	4510092	PP_4001	-	*crcB*, fluoride ion transporter crcB
D4	1114985	PP_0977	-	*valS*, valine-tRNA ligase
A5	4666235	PP_4129	+	*nuoL*, NADH-quinone oxidoreductase subunit L
B5	5012	PP_0005	-	*trmE*, GTPase
C5	2147244	PP_1905	-	*rne*, ribonuclease E
D5	953815	PP_0815	+	*cyoD*, cytochrome bo terminal oxidase subunit IV
A6	4667767	PP_4129	+	*nuoL*, NADH-quinone oxidoreductase subunit L
B6	564521	PP_0481	+	*katA*, catalase
C6	6037967	PP_5278	-	*kauB*, 4-guanidinobutyraldehyde dehydrogenase
D6	4661137	PP_4124	+	*nuoG*, NADH-quinone oxidoreductase subunit G
A7	4666235	PP_4129	+	*nuoL*, NADH-quinone oxidoreductase subunit L

B7	5341691	PP_4700	+	*panC*, pantothenate synthetase
C7	408018	PP_0339	+	*aceE*, pyruvate dehydrogenase E1 component
D7	4663844	PP_4125	+	*nuoH*, NADH-quinone oxidoreductase subunit H
B8	728512	PP_0623	+	*rluD*, 23S rRNA pseudouridine (1911/1915/1917) synthase
C8	838044	PP_0721	-	*rplY*, 50S ribosomal protein L25/general stress protein Ctc
8D	4661137	PP_4124	+	*nuoG*, NADH-quinone oxidoreductase subunit G
A9	406367	PP_0338	-	*aceF*, AceF-S-acetyldihydrolipoate
C9	175069	PP_23SA	+	PP_23SA, rRNA
D9	6114400	PP_5365	-	PP_5365, fatty acid methyltransferase
A10	6071679	PP_4326	-	*pstB-II*, phosphate ABC transporter ATP-binding protein
B10	4730997	PP_4187	-	*lpdG*, dihydrolipoyl dehydrogenase
C10	5354784	PP_4710	-	*truB*, tRNA pseudouridine(55) synthase TruB
D10	175007	PP_23SA	+	PP_23SA, rRNA
A11	5341691	PP_4700	+	*panC*, pantothenate synthetase
B11	182076	PP_0160	-	PP_0160, ferrioxamine receptor
C11	4739474	PP_4194	+	*gltA*, citrate synthase
D11	171389	PP_16SA	+	PP_16SA, rRNA
A12	5145431	PP_4532	-	*srmB*, ATP-dependent DEAD-box RNA helicase
B12	950646	PP_0812	+	*cyoA*, cytochrome bo terminal oxidase subunit II
C12	990120	PP_0856	+	PP_0856, lipoprotein
D12	5005032	PP_4410	+	PP_4410, hypothetical protein

Supplementary Table S3. Localization mini-Tn*5* [OPT•FILM] insertions in the genome of *P. putida* KT2440 *wsp*. Clones without a specific location or having a fraction of the pBAMD1.2 backbone were classified as illegitimate insertions. Genome coordinates and gene number from www.pseudomonas.com

**Clone**	**Genome coordinate**	**PP number**	**Strand**	**Gene name and putative function**
E1	950646	PP_0812	+	*cyoA*, cytochrome bo terminal oxidase subunit II
F1	4739122	PP_4193-PP_4194	none	Intergenic region between *gltA* and *sdhC*
G1	178707	PP_23SB	+	PP_23SB, rRNA
H1	175069	PP_23SA	+	PP_23SA, rRNA
E2	5546480	PP_4880	-	*rnr*, exoribonuclease R
F2	5669080	PP_4976	+	*ahcY*, adenosylhomocysteinase
G2	264770	PP_mr03	+	PP_mr03, ncRNA
H2	728512	PP_0623	+	*rluD*, 23S rRNA pseudouridine (1911/1915/1917) synthase
F3	141847	PP_0133-PP_0134	none	Intergenic region between PP_0134 and *algB*
G3	5340632	PP_4998-PP4699	none	Intergenic region between *folk* and *panB*
E4	4661137	PP_4124	+	*nuoG*, NADH-quinone oxidoreductase subunit G
F4	5012	PP_0005	-	*trmE*, GTPase
G4	5012	PP_0005	-	*trmE*, GTPase
H4	4661137	PP_4124	+	*nuoG*, NADH-quinone oxidoreductase subunit G
E5	802548	PP_0690	+	*obg*, GTPase
F5	868079	PP_0751	-	*mqo-I*, malate:quinone oxidoreductase
G5	8086479	PP_5339	+	*oruR*, ornithine utilization transcriptional regulator
H5	1151276	PP_1010	+	*edd*, phosphogluconate dehydratase
E6	5572917	PP_4903	+	*rsgA*, ribosome biogenesis GTPase RsgA
F6	5630471	PP_4947	+	*putA*, proline dehydrogenase/1-pyrroline-5-carboxylate dehydrogenase
G6	171389	PP_16SA	+	PP_16SA, rRNA
H6	324715	PP_0268	+	*oprQ*, outer membrane porin D
E7	4528401	PP_4017		PP_4017, hypothetical protein

G7	171389	PP_16SA	+	PP_16SA, rRNA
E8	2234890	PP_1974	+	*uvrB*, uvrABC excinuclease protein B
F8	6112217	PP_5363	-	PP_5363, hypothetical protein
G8	5486672	PP_4824	+	PP_4824, two-component system sensor histidine kinase/response regulator
H8	5781526	PP_5067	+	*kefA*, intermediate mechanosensitive channel protein
E9	307698	PP_0253	+	*pckA*, pseudogene
F9	175069	PP_23SA	+	PP_23SA, rRNA
H9	5646850	PP_4955	-	PP_4955, hypothetical protein
E10	6095282	PP_5347	-	*pycA*, pyruvate carboxylase subunit A
F10	6112871	PP_5364	+	*clsA*, cardiolipin synthase
G10	5012	PP_0005	-	*trmE*, GTPase
H10	5741075	PP_5038	-	PP_5038, lipoprotein
E11	408018	PP_0339	+	*aceE*, pyruvate dehydrogenase E1 component
F11	307164	PP_0253	+	*pckA*, pseudogene
G11	4739474	PP_4194	+	*gltA*, citrate synthase
H11	5953230	PP_5218	+	PP_5218, DedA family protein
E12	2486	PP_0004	-	*trmF*, tRNA uridine 5-carboxymethylaminomethyl modification protein
F12	5549759	PP_4881	+	PP_4881, iron ABC transporter substrate-binding protein
G12	5012	PP_0005	-	*trmE*, GTPase

Supplementary Table S4. Primers used in this study.

Oligo name	Sequence	Amplification
PleD-F	CATTTTTAAAAAGAGGAGAAATACT AGATGAGCGCCCGGATCCTCG	*pleD* gene
PleD-R	GCCTTTCGTTTTATTTGATGCCTTTAATCAGGCGGCCTTGCCGA CC	*pleD* gene
GSB9/GFP-F	TTAAAGGCATCAAATAAAACGAAAGG	pGreenL fragment 1
GSB9/GFP-R	CTAGTATTTCTCCTCTTTTTAAAAATG	pGreenL fragment 2
CcaSR-1R	CTAGCACTATACCTAGGACTG	pGreenL fragment 1
CcaSR-1F	ACCCCAGTTTTTACGGCTAG	pGreenL fragment 2
CcaSR/pBAM -F	TTGTGTCTCAGGCCGCCTAGGCCGCGGCCGCGCGAATTCACC CCAGTTTTTACGGCTAGCTC	CcaSR PleD cassette fragment 1
CcaSR-2R	CTCCTCTTTAACTAGCCTTCGGAGG	CcaSR PleD cassette fragment 1
CcaSR-2F	AAGGCAGCACCGAAGTTGG	CcaSR PleD cassette fragment 2
CcaSR/PleD/ pBAM-R	GAAAAGCCCGCCTTTCGGCGGGCTTTGGCGGCCGCAAGCTTG ATGCCTTTAATTAATCAGGCGG	CcaSR PleD cassette fragment 2
ARB6	GGCACGCGTCGACTAGTACNNNNNNNNNNACGCC	1st arbitrary PCR[10]
ARB2	GGCACGCGTCGACTAGTAC	2nd arbitrary PCR[10]
ME-O-ext-F	CGTCTGTTTCAGAAATATGGCAT	1st arbitrary PCR[10]
ME-I-ext-R	CTCGTTTCACGCTGAATATGGCTC	1st arbitrary PCR[10]
ME-O-int-F	ATCTGATGCTGGATGAATTTTTC	2nd arbitrary PCR[10]
ME-I-int-R	CAGTTTTATTGTTCATGATGATATA	2nd arbitrary PCR[10]

10 Primers for amplifying the boundaries of mini-Tn*5* [OPT•FILM] insertions in the genome of *P. putida* KT2440 (Martínez-García E, Aparicio T, de Lorenzo V, Nikel PI. New transposon tools tailored for metabolic engineering of gram-negative microbial cell factories. *Front Bioeng Biotechnol* **2:** 46, 2014)

Supplementary Fig. S1. Growth curves of KT2440 (top) and its *wsp* derivative (bottom) bearing genomic insertions of mini-Tn*5* [OPT•FILM] transposon.

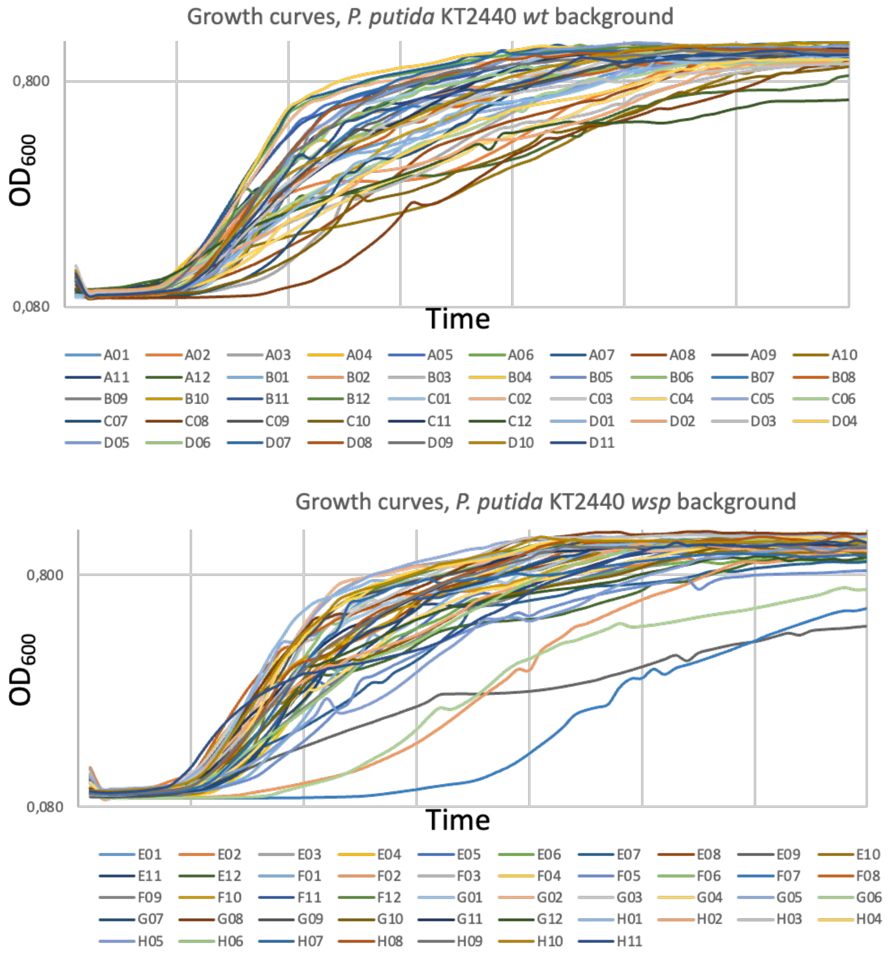

Graphs represent growth curves for each clone tested during 23 hours in 200 μL of LB medium at room temperature in 96-well microtiter plates, with 5 seconds of shaking before every measurement point (20 min) Strains were inoculated from an O/N pre-inoculum at an OD_{600} of 0.01 for three biological replicates.

Supplementary Fig. S2. Distribution of mini-Tn*5* [OPT•FILM] insertions through the genome of *P. putida* KT2440 and its *wsp* derivative.

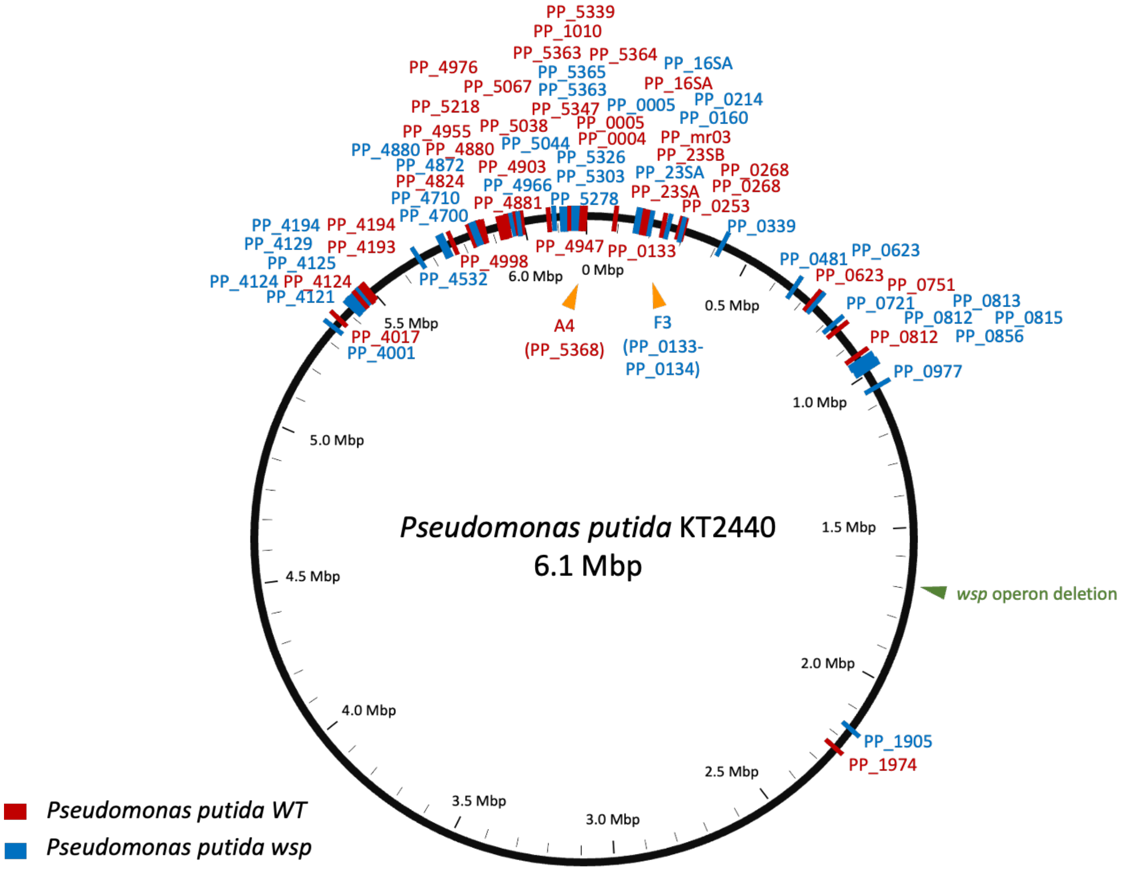

The picture summarizes insertions of the mobile element at different locations of the *P. putida* genome, either I the wild-type strain (red) or the *wsp* mutant (blue) as indicated. Note that with the transposon delivery protocol used most insertions occur in the portion of the chromosome closer to the origin of replication. Clones A4 (PP_5368) from *P. putida* wt background and F3 (PP_0133-PP_0134) from *P. putida* wsp background were also marked in the map as they were found to be the best performers.

REFERENCES

1. Grant SG, Jessee J, Bloom FR, Hanahan D. (1990) Differential plasmid rescue from transgenic mouse DNAs into *Escherichia coli* methylation-restriction mutants. *Proc Natl Acad Sci USA* 87, 4645-4649

2. Schmidt JJ. (1986) DNA Cloning: A Practical Approach. *Biochemical Education 14,* 91.

3. Nelson KE, *et al.* (2002) Complete genome sequence and comparative analysis of the metabolically versatile *Pseudomonas putida* KT2440. *Environ Microbiol 4,* 799-808.

4. Hueso-Gil Á, Calles B, de Lorenzo V. (2020) The Wsp intermembrane complex mediates metabolic control of the swim-attach decision of *Pseudomonas putida. Environ Microbiol 22,* 3535-3547.

5. Paul R, Abel S, Wassmann P, Beck A, Heerklotz H, Jenal U. (2007) Activation of the diguanylate cyclase PleD by phosphorylation-mediated dimerization. *J Biol Chem 282,* 29170-29177.

6. Aldridge P, Paul R, Goymer P, Rainey P, Jenal U. (2003) Role of the GGDEF regulator PleD in polar development of *Caulobacter crescentus. Mol Microbiol 47,* 1695-1708.

7. Hueso-Gil A, Nyerges Á, Pál C, Calles B, de Lorenzo V. (2020) Multiple-Site Diversification of Regulatory Sequences Enables Interspecies Operability of Genetic Devices. *ACS Synth Biol 9,* 104-114.

8. Martínez-García E, Aparicio T, de Lorenzo V, Nikel PI. Engineering Gram-Negative Microbial Cell Factories Using Transposon Vectors. *Methods Mol Biol 1498,* 273-293 (2017).

9. Kessler B, de Lorenzo V, Timmis KN. (1992) A general system to integrate *lacZ* fusions into the chromosomes of gram-negative eubacteria: regulation of the *Pm* promoter of the TOL plasmid studied with all controlling elements in monocopy. *Mol Gen Genet 233,* 293-301.

10. Martínez-García E, Aparicio T, de Lorenzo V, Nikel PI. (2014) New transposon tools tailored for metabolic engineering of gram-negative microbial cell factories. *Front Bioeng Biotechnol 2,* 46.
